# Recent Developments in Engineering Non-Paralytic Botulinum Molecules for Therapeutic Applications

**DOI:** 10.3390/toxins16040175

**Published:** 2024-04-03

**Authors:** Aisha Zhantleuova, Charlotte Leese, Anna P. Andreou, Altynay Karimova, Guy Carpenter, Bazbek Davletov

**Affiliations:** 1 Department of Biophysics, Biomedicine and Neuroscience, Al-Farabi Kazakh National University, Almaty A15E3C7, Kazakhstan; zhantleuova_aisha@live.kaznu.kz (A.Z.); altynai.karimova@kaznu.edu.kz (A.K.); 2 Department of Biomedical Science, University of Sheffield, Sheffield S10 2JA, UK; c.leese@sheffield.ac.uk; 3 Headache Research, Wolfson Centre for Age-Related Diseases, Institute of Psychiatry, Psychology and Neuroscience, King’s College London, London SE1 1UL, UK; anna.andreou@kcl.ac.uk; 4 Neuresta, Inc., San Diego, CA 91991, USA; 5 Salivary Research, Centre for Host-Microbiome Interactions, Faculty of Dental, Oral & Craniofacial Sciences, King’s College London, London SE1 1UL, UK; guy.carpenter@kcl.ac.uk

**Keywords:** botulinum neurotoxin, treatment, non-paralytic molecules, efficacy, safety

## Abstract

This review discusses the expanding application of botulinum neurotoxin in treating neurological conditions. The article specifically explores novel approaches to using non-paralytic botulinum molecules. These new molecules, such as BiTox or el-iBoNT, offer an alternative for patients who face limitations in using paralytic forms of botulinum neurotoxin due to concerns about muscle function loss. We highlight the research findings that confirm not only the effectiveness of these molecules but also their reduced paralytic effect. We also discuss a potential cause for the diminished paralytic action of these molecules, specifically changes in the spatial parameters of the new botulinum molecules. In summary, this article reviews the current research that enhances our understanding of the application of new botulinum neurotoxins in the context of common conditions and suggests new avenues for developing more efficient molecules.

## 1. Introduction

Botulinum neurotoxins (BoNTs), the most potent paralytic agents known, have emerged as multipurpose cosmetic and therapeutic treatments, with more applications in modern medicine than any other drug currently on the market. Initially developed for the treatment of strabismus and neurologic movement disorders [1,2], the use of BoNTs has been expanding during the past 3 decades, with current therapeutic and cosmetic applications making many billions of USD a year. Clinical applications now include the treatment of a variety of ophthalmologic, gastrointestinal, urologic, orthopedic, dermatologic, oral, secretory, pain, and other conditions [3,4,5,6,7,8,9,10].

## 2. Mechanisms of Action of BoNTs

Understanding the mechanisms underlying the action of BoNTs has been instrumental in their diverse therapeutic applications. These mechanisms can be categorized into three main pathways: neuromuscular junction modulation, modulation of excretory glands, and nociception regulation.

The primary mechanism of BoNT action involves the relaxation of hyperactive muscles through their interaction with the peripheral neuromuscular junction. BoNTs block cholinergic neurons, thereby inhibiting the release of acetylcholine and disrupting neural transmission. This interference leads to the relaxation of muscles, making BoNTs effective in treating conditions characterized by muscle spasms [11]. BoNTs also demonstrate efficacy in modulating the hyperfunction of certain excretory glands by inhibiting the release of acetylcholine from nerve endings. At the neuromuscular junction, BoNTs induce muscle tone loss, and in glandular tissues, they inhibit cholinergic sympathetic nerve function [12]. The analgesic effects of BoNTs, though not fully explained, are thought to occur through multiple mechanisms. Firstly, BoNTs inhibit the release of pain neurotransmitters such as substance P (SP), calcitonin gene-related peptide (CGRP), and glutamate, which are involved in pain signal transmission [13,14,15]. The second proposed mechanism for the analgesic effect of botulinum drugs is the modulation of ion channel expression in nociceptors [16], potentially reducing and normalizing the levels of receptors such as TRPA1, TRPV1, and P2X3 [17,18,19]. Furthermore, BoNTs may have a third antinociceptive mechanism which is related to its effect on the central nervous system, with evidence indicating the axonal transport of BoNTs to sensory nociceptive nuclei [20].

## 3. Clinical Use of BoNTs

The current understanding of botulinum neurotoxins’ mechanisms enables their application not only in conditions necessitating muscle relaxation, such as dystonias, blepharospasm, etc., but also in external gland hypersecretion and pain syndromes, which is the topic of this review.

### 3.1. Efficacy of BoNTs in External Gland Secretion

BoNT therapy is effective in reducing external gland secretion and alleviating the associated symptoms across various autonomic disorders. Clinical studies have demonstrated significant improvements in sweat production, salivary flow, tear secretion, and sebum production following BoNT injections.

#### 3.1.1. Hyperhidrosis

Hyperhidrosis is a common disorder where uncontrollable excessive sweating affects the axillae, palms, soles of the feet, and face. Although BoNT is widely used for many types of hyperhidrosis, it was only approved by the Food and Drug Administration (FDA) as a treatment for primary axillary hyperhidrosis in 2004 [21]. Studies showed that the treatment of hyperhidrosis is both effective and safe, lasting up to approximately 9 months [22]. Another study confirmed the efficacy of the treatment with 87% of patients satisfied with the treatment and 74% of patients continuing follow-up treatment for 2 years with a periodicity of one injection every 5–6 months [23]. However, the use of this toxin is limited due to its paralytic properties, especially when treating the craniofacial area. Unfortunately, despite its high efficacy in reducing sweating and long duration of action, BoNT injections lead to the paralysis of the frontalis muscle in 50–100% of cases [24]. Side effects also included stiffness of the forehead and the eyebrows.

#### 3.1.2. Sialorrhea

Several neurodegenerative diseases such as Parkinson’s disease, amyotrophic lateral sclerosis, and infant cerebral palsy can lead to a reduced ability to swallow saliva, resulting in sialorrhea or drooling which dramatically affects the lives of patients [21]. In recent years, injections of BoNT into salivary glands was shown to be effective in reducing saliva flow without systemic side effects, both in adults and children [25,26]. BoNT injections were approved for managing sialorrhea in 2019 [27]. While adverse events related to BoNT injections are rare, it is noteworthy that severe dysphagia can occur in children, leading to compromised food and fluid intake, as well as weight loss. Additionally, xerostomia, dysarthria, speech difficulties, coughing, and feeding difficulties have been reported, along with instances of saliva thickening and weakening of the oral musculature [28]. In the study by Yu et al., among adults, adverse events were reported in 7 out of 12 trials. Dry mouth occurred more often in the BoNT group compared to the placebo group, but there was no significant difference in dysphagia occurrence. Some patients in the BoNT group had treatment-related pneumonia [29].

#### 3.1.3. Hyperlacrimation

Recent research indicates that BoNT injections into the lacrimal gland can effectively treat hyperlacrimation. This approach works by chemodenervating the cholinergic neurons of the parasympathetic nervous system that serve the lacrimal gland, thereby diminishing tear production. Clinical studies have demonstrated that patients experienced relief from symptoms, with some reporting a recurrence approximately 3.5 months post-treatment, while others remained symptom-free up to the 6-month evaluation. However, this treatment does come with potential side effects such as ptosis, dry eye, and an increased awareness of the eye that received treatment [30,31]. Higher doses of BoNT/A have been linked to an increased incidence of local complications [32]. This approach has been also applied to manage epiphora (excessive tearing) with no significant systemic adverse reactions reported. However, the complications were generally mild, including temporary non-obstructive ptosis and isolated instances of diplopia and esotropia, which were transient [33].

#### 3.1.4. Facial Seborrhea

BoNT is being effectively used in treating oily skin and seborrheic dermatitis through reducing sebum production. This reduction is thought to result from BoNT’s ability to interrupt nerve signals that control sebum output. Clinical studies have demonstrated that BoNT injections can significantly diminish skin oiliness and shrink pore size, with patients observing an up to 80% decrease in oiliness after one month [34]. These treatments have also led to smoother skin and fewer expression lines, all without notable side effects [35].

### 3.2. Efficacy of BoNT in Hypersensitivity

The use of BoNT in the treatment of different hypersensitivity disorders is growing.

#### 3.2.1. Overactive Bladder

BoNT, when utilized as a therapy for overactive bladder (OAB), is believed to obstruct nerve signals responsible for triggering OAB symptoms, thereby offering symptomatic relief. Indeed, in the treatment of OAB with BoNT, it is important to note that its action is not solely attributed to muscle relaxation but also involves blocking sensitive receptors [36]. Currently, the injection of BoNT/A is approved by the FDA as a third-line therapy for OAB [37]. Studies have shown that BoNT/A therapy leads to significant improvements in urinary symptoms for up to 12 weeks [38]. When treating OAB with BoNT, it is important to be aware of the potential adverse effects that may occur, which can include a higher incidence of urinary tract infections (UTIs) and voiding difficulties necessitating self-catheterization [39,40]. The need of self-catheterization occurs due to excessive detrusor relaxation following injections, as highlighted by the studies conducted by Cui et al. and Zhou et al. [41,42]. Men exhibit more than 2 times higher odds of incomplete emptying compared to women, and following injection, 17% of men and 23.5% of women encounter more than one episode of a UTI within the initial month [43].

#### 3.2.2. Pain Syndromes

BoNT has demonstrated efficacy in alleviating various types of pain, including neuropathic pain and chronic pain associated with specific conditions. Studies have shown its effectiveness in conditions such as trigeminal neuralgia (TN), post-herpetic neuralgia, painful diabetic neuropathy, central neuropathic pain in multiple sclerosis, pain in traumatic brain injury/spinal cord injury, and post-stroke pain [44]. BoNT injections have also been used to treat pelvic pain, urological pain, and pain associated with cancer, resulting in significant pain reductions and improvements in quality of life in some cases [45,46].

##### Chronic Migraine

Currently, the only pain condition that has FDA approval for treatment using BoNT is chronic migraine, which is a complex neurological disorder thought to be initiated by trigeminal nociceptor activation [16]. Several clinical trials and meta-analyses have shown that botulinum injections effectively reduce the frequency, severity, and duration of migraine headaches in patients with chronic migraine. These studies indicate that the treatment can lead an average decrease of 7.6 to 8.7 migraine days per month, a cumulative decrease of 106.7 to 132.4 headache hours on headache days, and improvements of 4.7 to 4.9 points in the HIT-6 score [47,48,49]. The results of clinical trials showed high efficacy in patients in whom other migraine treatments had been ineffective. Unfortunately, only 50% of patients report some relief from migraine after BoNT injections around the scalp area [50]. Also, side effects such as temporary weakness or paralysis of nearby muscles are associated with using BoNT [51]. Based on the available data, it has been observed that about 60 out of 100 participants reported a side effect when using BoNT injections. Eyelid drooping and muscle weakness were reported as the most common side effects [52].

##### Trigeminal Neuralgia

BoNT/A is typically used in cases of TN that do not respond to conventional oral therapies. However, despite its effectiveness, approximately 10–43% of patients treated with BoNT for TN still do not experience relief from their symptoms [53]. BoNT has been found to be a safe and effective treatment for TN, with temporary facial asymmetry being the most common adverse event. This side effect is generally mild to moderate and typically resolves within 2–3 weeks to 1–2 months [54]. Additional adverse effects of BoNT treatment for TN include edema, hematoma, pain, and masticatory disturbances [55].

## 4. Adverse Effects

Botulinum therapy can lead to adverse events, which may arise from an individual’s physiological reactions, such as allergic responses, and the technique of drug administration, which includes considerations like the drug’s dose, its dilution, and the choice of injection site. Furthermore, the intrinsic effects of the drug can cause undesirable outcomes like muscle relaxation, both localized and general, and dryness of the eyes, among others. These adverse effects might also emerge from the interplay of various factors, for example, ptosis or eye dryness due to the incorrect dose or injection site in the treatment.

To categorize the side effects, they are divided into those unrelated to the myorelaxant action and those that are a direct consequence of it (Table 1).

Adverse effects not related to the paralytic action of botulinum toxin are usually not serious and pass quickly. Adverse effects associated with the paralytic action of the drug are more serious and limit the use of botulinum toxin. The development and introduction of botulinum toxins without paralytic action, but maintaining the effect on sensitive receptors and excretion, will expand the possibilities for the wider use of botulinum toxin.

## 5. Novel BoNT Molecules

A literature search was conducted using the PubMed database for the period from January 2003 to December 2023. The search included the following keywords used in different combinations: “botulinum neurotoxin”, “non-paralytic molecules/nonparalytic molecules”, “synthetic toxin”, and “engineered molecules”. Manual study searches were then performed using the reference lists of the articles found during the search process. Six articles were ultimately chosen for review. This process involved assessing the articles for their focus on the development of non-paralytic botulinum molecules for therapeutic applications, language suitability (English), peer-reviewed status, and original research content.

**Figure 1:**
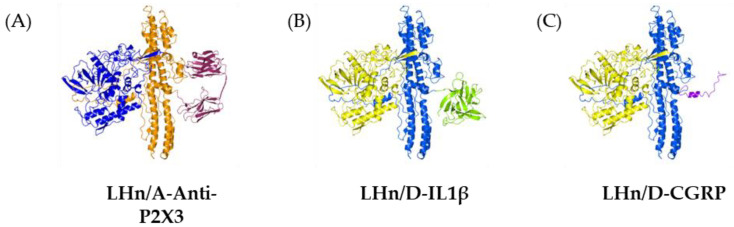
Structural representations of engineered botulinum neurotoxin constructs: (**A**) LHn/A-Anti-P2X3 (light chain/A: blue; translocation domain/A: orange; ScFv: purple), (**B**) LHn/D-IL1β (light chain/D: yellow; translocation domain/D: blue; IL1β: light green), (**C**) LHn/D-CGRP (CGRP: light purple). CCP4MG software (version 2.10.11) was used to create the 3D models. Structures used were BoNT/A from PDB 3BTA [59]; ScFV from PDB 4OUO [60]; BoNT/D from PDB 5BQN [61]; IL1B from PDB 1HIB [62]; and CGRP from PDB 7TYO [63].

**Figure 2:**
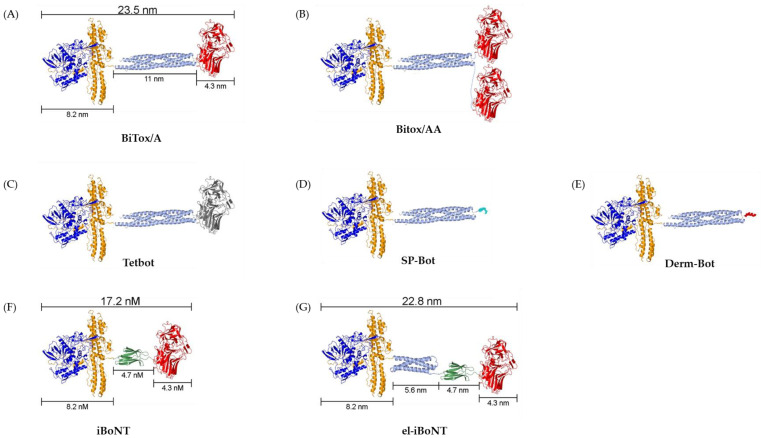
Comparisons of botulinum neurotoxin (light chain: blue; translocation domain: orange; receptor-binding domain: red) constructs: (**A**) non-paralytic BiTox/A (SNARE helix: light blue), (**B**) BiTox/AA, (**C**) TetBot (receptor-binding domain of the tetanus toxin: grey), (**D**) SP-Bot (Substance P: turquoise), (**E**) Derm-Bot (dermorphin peptide: red), (**F**) paralytic iBoNT (SpyCatcher–SpyTag: green), (**G**) non-paralytic elongated iBoNT (syntaxin-derived extension sequence: light blue). CCP4MG software (version 2.10.11) used to create the 3D models. Structures used were BoNT/A from PDB 3BTA [59]; SNARE complex from PDB 1SFC [64]; syntaxin extension sequence from PDB 1EZ3 [65]; SpyCatcher–SpyTag from PDB 4MLI [66]; and substance P from PDB 7RMH [67].

Research is underway to develop new non-paralytic botulinum molecules and evaluate their safety and efficacy to reduce the likelihood of the previously discussed side effects, thereby increasing the doses that can be used for more efficient treatments. Researchers are using various re-engineering techniques to eliminate the paralytic activity of BoNT/A and redirect its binding specifically to sensory neurons. These include traditional methods such as recombinant protein expression and chemical conjugation (Figure 1) and more recent methods such as SNARE-stapling and SpyCatcher–SpyTag technology (Figure 2).

One of the methods used for protein production is recombinant expression. In a study by Ma and colleagues, the gene encoding the receptor-binding domain of BoNT/A was deliberately omitted. Instead, the gene encoding an antibody against P2X3, a receptor specific for nociceptive neurons, was combined with the gene encoding BoNT/A endopeptidase. The resulting protein (Figure 1A) can specifically bind to cultured neurons expressing P2X3 and retains BoNT/A endopeptidase activity, resulting in synaptosomal-associated protein of 25 kDa (SNAP-25) cleavage and reduced CGRP release from these neurons. One of the key advantages of this reconstructed protein is its significantly improved safety profile compared to the native BoNT/A. This protein can potentially be used at higher doses without undesirable side effects [68]. It is important to note that this study was performed in vitro, and further studies are required to evaluate the safety and efficacy of this modified protein in vivo.

Another approach to protein modification is represented by chemical conjugation. For example, to obtain a chimera capable of inhibiting the transmission of pain sgnals, maleimide conjugation was used to bind the light chain of BoNT/A protein to SP, whichis involved in the transmission of pain signals [69]. In a study by Tang et al. [70] two new types of BoNT (Figure 1B,C) were developed using sortase A-based technology. IL1β ligands and the CGRP receptor antagonist were attached to translocation domain and the light chain of the BoNT/D, which results in the cleavage of vesicle-associated membrane protein (VAMP) to block secretion. The resulting modified BoNTs were able to penetrate macrophages and dorsal medullary ganglion neurons and stop the release of the inflammatory mediators IL6 and SP, which is mediated by VAMP cleavage.

Despite the promise of re-engineering the BoNT/A protein using recombinant expression and chemical conjugation for targeting specific pain pathways, both methods have their limitations. Recombinant expression can be limited by large protein sizes and misfolding problems, while there are doubts about the long-term stability of chimeras created by chemical conjugation. It is also unclear if other, non-target non-neuronal cells carrying receptors for the ligands used (P2X3 antibody, CGRP, IL1) could be affected by the chimeric botulinum molecules and bring about non-desired side effects. In addition, as mentioned above, the efficacy of these BoNT/A chimeras in treating pain has yet to be rigorously tested in vivo, and it remains to be seen whether they will prove effective in clinical settings. However, these approaches hold a potential for developing new analgesic drugs that could provide much-needed relief for patients suffering from chronic pain.

Recently, researchers have developed a new protein engineering method called “protein stapling” technology as a solution to the shortcomings of recombinant expression and chemical conjugation. This method combines elements of recombinant protein expression with peptide linking. It is based on the properties of SNARE complex proteins, such as VAMP, syntaxin, and SNAP-25, which can self-assemble into a tetrameric spiral. To create hybrid molecules, a SNAP-25 linker was attached to the protease and translocation domains of BoNT/A and the VAMP linker was attached to the receptor-binding domain. When the stapling peptide syntaxin was added, the SNARE proteins self-assembled, and the three botulinum domains combined to form a single functioning drug, which was called BiTox, short for Binary Toxin (Figure 2A) [71]. BiTox was demonstrated to have activity similar to that of the native drug in neuronal cultures. This indicates that SNARE “cross-linking” does not affect the translocation and proteolytic activity of the corresponding parts of the toxin. Importantly, the effectiveness of BiTox in blocking neuromuscular junctions was lower, probably due to the reduced ability of the structurally extended toxin to enter small synaptic vesicles in motor neurons or to reach active zones. The increased size of BiTox allows it to block sensory pathways involved in pain transmission without causing local neuromuscular paralysis [72]. Indeed, the main difference between the native toxin and BiTox is that “cross-linking” almost doubles the drug’s size [71]. A study by Mangione et al. [73] also demonstrated that BiTox is an effective analgesic for treating neuropathic pain. It induces a long-term reduction in mechanical hyperalgesia, which was evident after three days of use.

The “stapled” botulinum neurotoxin molecule has one further advantage as it is safe to produce because its components are non-functional and, therefore, non-toxic before assembly with the linker syntaxin peptide. The flexible assembly of different subunits is also possible. Receptor-binding domains can be duplicated to enhance cell targeting efficiency, and the native receptor-binding domain can be replaced with other ligands to target different cell types.

A study by Andreou et al. [74] studied a new botulinum molecule designed to reduce the muscle-paralyzing properties for potential use in the treatment of chronic migraine. This molecule, called binary toxin-AA (BiTox/AA) (Figure 2B), contains a duplicated binding domain that promotes neuronal binding and the cleavage of SNAP-25 with an efficacy comparable to that of BoNT/A. In contrast to BoNT/A, BiTox/AA shows little paralytic effects on muscle, as confirmed by compound muscle action potential recordings after injection into the calf muscle. The paralytic effect of BiTox/AA is 100 times less than that of the native BoNT/A.

Another chimeric molecule cross-linked from the light chain and translocation domains of BoNT/A ‘’stapled” to the receptor-binding domain of the tetanus toxin (Figure 2C) has also been created. Normally tetanus toxin is internalized, via motor neurons, into spinal cord inhibitory neurons, leading to VAMP cleavage in these neurons and spastic paralysis. As would be expected from an elongated botulinum construct, the new tetanus–botulinum chimera (TetBot) was non-paralytic but efficiently cleaved SNAP-25 in central nervous system neuronal cultures. When delivered intrathecally, this toxin can reduce the mechanical sensitivity in a Freund’s Complete Adjuvant (CFA)-induced inflammation model but does not cause paralysis, either flaccid or spastic [75]. The widespread immunization against tetanus toxin in humans limits the potential utility of TetBot for human medical care. However, in the context of animal health, specifically in veterinary medicine, TetBot may offer a viable option for pain management.

Using similar stapling technology, two peptide–botulinum preparations have been created, named substance P-Bot (SP-Bot) (Figure 2D) and Dermorphin-Bot (Derm-Bot) (Figure 2E). SP-Bot consists of an enzymatic and translocation BoNT/A domain linked to the substrate P ligand. This targeting domain enables the internalization and suppression of neurokinin 1 receptor-expressing neurons in the spinal cord that transmit pain signals. Intrathecal injection of this construct reduces mechanical hyperalgesia in established pain conditions, including CFA-induced peripheral inflammation and neuropathy caused by experimental nerve damage in the leg. Derm-Bot is also a BoNT/A-based conjugate linked to the dermorphin peptide as its opioid receptor-binding domain. When this construct is injected intrathecally, spinal cord neurons that express mu-opioid receptors are suppressed, providing an effect similar to traditional opiates albeit with doses which are 1000 times smaller than opiates. Derm-Bot injections can reverse the hyperalgesia induced by experimental nerve damage with an efficacy equivalent to morphine injections but lasts for at least 25 days in mice after a single injection [76].

SpyCatcher–SpyTag technology [77] is a novel method of creating hybrid botulinum drug molecules with a reduced paralytic effect. The method is based on the use of a bacterial module called SpyCatcher and a short peptide tag called SpyTag that are used to link any two proteins by forming an isopeptide bond (Figure 2F). SpyCatcher and SpyTag can be added to different domains of botulinum proteins and then these components can be linked by forming a permanent bond between them. This approach was tested in a recent article by Leese [78]. The study examined the analgesic potency and paralytic effect of a modified botulinum molecule called “elongated isopeptide-bonded” BoNT (el-iBoNT) (Figure 2G). The researchers tested its effectiveness in neuronal cultures and rat models. They found that the elongated el-iBoNT exhibited potent SNAP-25 cleavage activity in sensory neurons, like native BoNT/A, indicating that it was functional. In rat models, el-iBoNT demonstrated reduced motor deficits in comparison with BoNT/A, indicating a reduction in its paralytic action. For its potential analgesic action, el-iBoNT was evaluated in a rat model of neuropathic pain. Animals injected with el-iBoNT exhibited a significant reduction in mechanical hypersensitivity compared to the control group, indicating pain relief. Furthermore, the observed analgesic effect of el-iBoNT was maintained over a prolonged period of time, with the animals exhibiting a mechanical sensitivity comparable to intact rats. These results show that el-iBoNT has analgesic properties without the paralytic side effects, making it a potential candidate for long-sought-after blockers for the treatment of chronic pain syndromes.

## 6. Future Directions

The paralytic botulinum neurotoxins are currently used to treat conditions that do not require their paralytic activity, including external gland hypersecretion, hypersensitivity, and pain syndromes. Despite the proven efficacy of this approach, there are limitations associated with the undesirable side effects linked to muscle paralysis, possible diffusion risk, and risk of stimulation of antibody formation.

The adoption of engineered non-paralytic botulinum molecules for therapeutic purposes offers a promising strategy to counteract the side effects linked to the paralytic action of traditional botulinum neurotoxins. The rationales for avoiding muscle paralysis include either retargeting the botulinum enzymatic activities away from neuromuscular junctions, as evidenced by the chimeric botulinum molecules, or elongating the botulinum molecules such that their entry into small synaptic vesicles is inhibited. Additionally, elongated botulinum constructs could still affect the open sensory nerve endings while their entry into the tight neuromuscular junctions is compromised. The utilization of engineered non-paralytic botulinum molecules for therapeutic purposes is a promising approach to also mitigate diffusion-related risks, as these toxins, even when spread out, do not exert paralytic effects. The new engineered non-paralytic botulinum molecules serve as prototypes for designing future therapeutics, highlighting the potential for tailored interventions with improved safety profiles.

The risk of stimulation of antibody formation is a challenge in the development of protein therapeutics. This is especially true for BoNT molecules. Studies have shown a correlation between increasing therapeutic doses of BoNT and enhanced immunity leading to the development of neutralizing antibodies [79]. Moreover, repeated exposure to BoNT can lead to clinical resistance due to antibody production as well [80]. The presence of protein complexes in commercially available BoNT preparations increases the risk of antigenicity. This increases the risk of treatment failure [81]. However, there are studies that emphasize the immunogenicity of BoNTs by selecting highly purified BoNT products early in treatment. This not only improves long-term outcomes and patient satisfaction, but it also reduces the risk of immune system activation and antibody neutralization [82]. In addition, there is an opportunity to diminish the risk of stimulation of antibody formation by using BoNTs with increased clinical efficacy and lowering the repetitively administered BoNT protein dosage [83]. This strategy is expected to delay the development of antibodies to BoNTs in patients and reduce the risk of antibody activation, while providing therapeutic efficacy.

To achieve therapeutic efficacy using non-paralytic BoNTs that are typically administered through injections at substantially higher doses, the potential risks associated with induction of an immune response must be carefully considered. Moreover, it is worth noting that the amplification of the administered dose of BoNTs aligns with an increased vulnerability to antibody formation. A thorough preclinical and clinical evaluation is required to fully assess the immunogenic potential of the engineered BoNT molecules. This aspect remains to be explored due to the novelty of the approach.

The potential for traditional botulinum neurotoxins to diffuse from the injection site into the bloodstream and potentially causing systemic effects, particularly on respiratory muscles, is a notable concern within clinical practice. This risk has been extensively documented in the literature, with instances of botulism-like generalized weakness and systemic muscular effects reported following the administration of botulinum neurotoxins [84,85]. It is hoped that the increased size of elongated botulinum molecules will decrease the risk of diffusion based on more favorable physico-chemical characteristics and this will need to be investigated in future studies.

The development of new non-paralytic botulinum molecules not only offers new possibilities for expanding the therapeutic potential of this treatment beyond its traditional use as a neuromuscular blocker, but it also allows for an increase in the maximum dose and decreased side effects. The new, non-paralytic molecules can provide desirable therapeutic effects without the risk of paralytic side effects, making botulinum drugs a more attractive option for the treatment of many conditions. Overall, the use of botulinum neurotoxin-based drugs is a promising area for further research and development. New engineered botulinum variants present a promising approach as a treatment option for chronic neurological conditions due to their long-lasting effects with minimal side effects.

## Figures and Tables

**Table 1:** The most common side effects related to use of botulinum neurotoxin for hypersecretion of externa glands, OAB, and pain syndromes.

Indication	Adverse Effects Not Related to Myorelaxant Action	Adverse Effects Related to Myorelaxant Action
Hyperhidrosis [56]	Anhidrosis Pain Bruising	Muscle weakness Facial asymmetry (facial hyperhidrosis) Handgrip weakness (palmar hyperhidrosis)
Sialorrhea [27]	Pain Dry mouth Viscous saliva	Tongue control Chewing weakness
Overactive bladder [57]	Pain Procedure-related urinary tract infections Mild hematuria	Urinary retention or intermittent self-catheterization
Chronic migraine [52]	Pain	Blepharoptosis Muscle weakness
Trigeminal neuralgia [58]	Hematoma Itching Pain Transient edema	Facial asymmetry

## List of Abbreviations

BoNT(s)	Botulinum Neurotoxin(s)
SP	Substance P
CGRP	Calcitonin Gene-Related Peptide
TRPA1	Transient Receptor Potential Ankyrin 1
TRPV1	Transient Receptor Potential Vanilloid 1
P2X3	P2X Purinoceptor 3
FDA	Food and Drug Administration
OAB	Overactive Bladder
UTI	Urinary Tract Infections
TN	Trigeminal Neuralgia
HIT-6	Headache Impact Test
SNARE	Soluble N-Ethylmaleimide-Sensitive Factor Attachment Protein Receptor
SNAP-25	Synaptosomal-Associated Protein of 25 kDa
IL1β	Interleukin-1β
VAMP	Vesicle-Associated Membrane Protein
IL6	Interleukin 6
BiTox	Binary Toxin
BiTox/AA	Binary Toxin-AA
TetBot	Tetanus-Botulinum Chimera
CFA	Freund’s Complete Adjuvant
SP-Bot	Substance P-Bot
Derm-Bot	Dermorphin-Bot
el-iBoNT	Elongated Isopeptide-Bonded Botulinum Neurotoxin
